# The Database for Reaching Experiments and Models

**DOI:** 10.1371/journal.pone.0078747

**Published:** 2013-11-14

**Authors:** Ben Walker, Konrad Kording

**Affiliations:** 1 Departments of Physical Medicine and Rehabilitation, Physiology, and Applied Mathematics, Northwestern University, Chicago, Illinois, United States of America; 2 Sensory Motor Performance Program, Rehabilitation Institute of Chicago, Chicago, Illinois, United States of America; SUNY Downstate MC, United States of America

## Abstract

Reaching is one of the central experimental paradigms in the field of motor control, and many computational models of reaching have been published. While most of these models try to explain subject data (such as movement kinematics, reaching performance, forces, etc.) from only a single experiment, distinct experiments often share experimental conditions and record similar kinematics. This suggests that reaching models could be applied to (and falsified by) multiple experiments. However, using multiple datasets is difficult because experimental data formats vary widely. Standardizing data formats promises to enable scientists to test model predictions against many experiments and to compare experimental results across labs. Here we report on the development of a new resource available to scientists: a database of reaching called the Database for Reaching Experiments And Models (DREAM). DREAM collects both experimental datasets and models and facilitates their comparison by standardizing formats. The DREAM project promises to be useful for experimentalists who want to understand how their data relates to models, for modelers who want to test their theories, and for educators who want to help students better understand reaching experiments, models, and data analysis.

## Introduction

Reaching is one of the popular paradigms used to study movement. Reaching is used to ask psychological, computational, behavioral, and clinical questions. For example, it is used to ask how people determine ownership of their hand [1], how the brain deals with uncertainty [2], how people minimize their variability [3], and how recovery of motor function after stroke can be accelerated [4]. In all these domains, the contributions of reaching experiments are important, both conceptually and practically, as their ramifications extend well beyond the domain of reaching.

Although reaching research is driven by a broad community, publications about reaching tend to be insular. These publications generally present one experiment or one model – or in some cases, one of each. However, comparing datasets or models from multiple publications can lead to new insights. For instance, comparing datasets can help identify relevant differences in experimental designs. Comparing models promises to reveal which aspects of models are most important. And comparing multiple datasets and models promises to expose the scope and limitations of each model. Data sharing – making models and experimental data publicly available – would facilitate these comparisons. In addition, data sharing could help assimilate new members into the reaching community. For instance, educators could enhance the learning experience by having students analyze data from recent publications. Access to experimental datasets and models would also reduce the barrier to entry for scientists studying other fields who want to transition into studying reaching. Sharing of data and models would stimulate productive scientific communication and facilitate new scientific inquiries.

Data sharing could be enabled by a database. However, data formats vary widely between labs – and sometimes even within the same lab. If a user of a database were required to learn a new format for each dataset or model, the database would be too cumbersome. But if a database were to use a standard format for datasets and another standard format for models, comparisons and model fits could be efficiently performed across a variety of datasets. Using common formats could simplify explanations of experiment results, allow for quicker evaluation of new models, and reduce, if not fully eliminate, problems of interpreting formats or variable names.

A consistent data format for reaching experiments is achievable because a significant portion of recorded data is similar across experiments. For example, tracking the hand in space is integral for reaching experiments. In the same way, many experiments involve some sort of perturbation. These perturbations can be visual, such as cursor feedback on screen (e.g. [5], which is in the DREAM database), or physical, such as movement through a force field (e.g. [6], which is in the DREAM database). A well-chosen data format could represent many experiments by taking advantage of these similarities. Likewise, models can be structured to follow one common format, accepting arguments in a standard way. Since models make predictions in the same space as experimental data, predictions can be stored in the same format as experimental data. Through standardization, the data/model comparison would then be straightforward; the process would remain unchanged iteration after iteration, regardless of which models or which experiments were used. Lastly, if the database were curated, such formats could be enforced for all datasets and models. Data could be shared with a curator who could put the data into the correct format before making it available for download.

**Figure 1:**
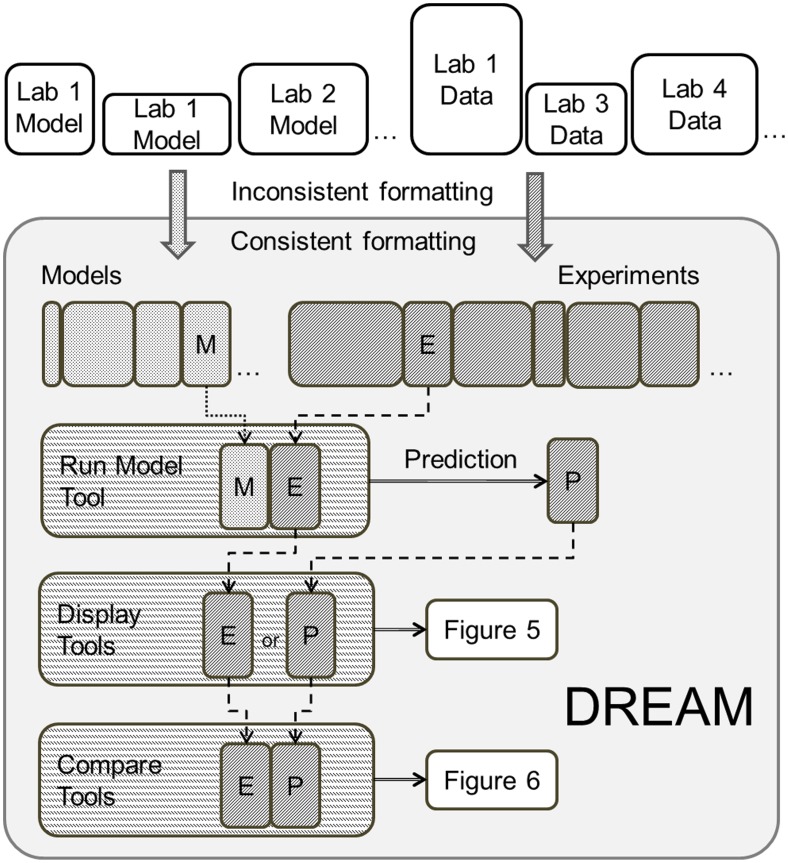
DREAM flow chart. Models and experimental data are collected from different labs and aligned to a common format that allows analyses using DREAM tools. Lighter solid boxes indicate a standard model format, darker solid boxes indicate a standard data format (for experimental data and model predictions), and striped boxes indicate DREAM tools. Because the model and data formatting is consistent throughout DREAM, tools can work for any model, experimental dataset, or model prediction.

Here we present such a database, the Database for Reaching Experiments And Models (DREAM). DREAM is a curated database that uses one format for many distinct experiments and model predictions and another format for models. A user can download DREAM datasets, models, tools, and documentation from http://crcns.org/data-sets/movements/dream/. We chose MATLAB as the development environment due to its popularity in the field; as such, the files associated with the database are .mat and .m files. Importantly, through data standardization, generic tools can be written that will generally work with every experimental dataset and every model in the database. The user could load any dataset into MATLAB, use a DREAM tool to run a model and generate predictions, then use other tools to display and compare predictions and experimental data (see Fig. 1). We will briefly describe the format and reasoning behind the content of DREAM (fuller documentation can be found online), then show how the tools can be used to display data, compare model predictions against experimental data, and compare model predictions against one another.

## Materials and Methods

**Figure 2:**
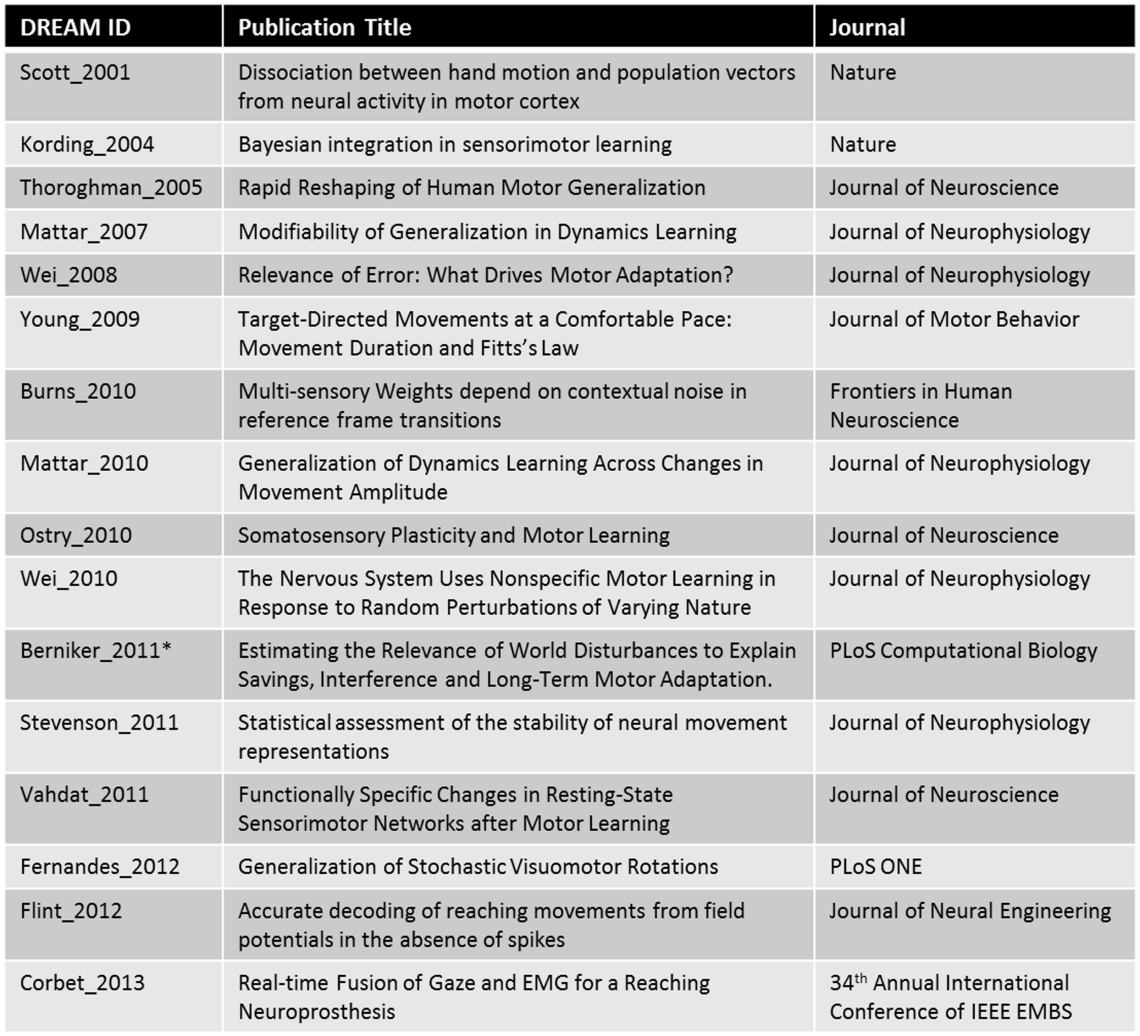
Data in DREAM. Publications from which data has been added to DREAM. The DREAM ID is a unique identifier created by combining the last name of the first author and the year of publication.

Datasets and models were acquired from many scientists in the reaching field. Currently, DREAM has data from 16 different publications (see Fig. 2), citing author affiliations with 15 different institutions; 39 experiments and 5 models have thus far been added into DREAM. Willingness to share data has been shown to be an indicator of good science [7], so we are confident in the quality of these studies.

### Experiments

We use one format to represent many unique experiments. DREAM contains experiments that alter how the subject receives visual feedback; a cursor (or “cloud of dots” for uncertainty experiments) is displayed continuously [8], only at the midpoint [9], or only at the target [10]. Some experiments change reaching environments from trial to trial, alternating force fields and catch trials [2]. Other experiments involve visuomotor rotations [5], learning of force fields [6], generalizing reach distances [11] and directions [12], changing the movement pace [13], and reference frame transformation [14]. DREAM also has datasets from non-human primates that recorded local field potentials [15], neuron spike timestamps [16] and behavior but not brain activity [17]. We also have experiments that include important non-behavioral data, such as recorded functional magnetic resonance imaging (fMRI) scans of subjects before and after certain movement conditions [18] or recorded electromyography (EMG) activity of four shoulder muscles [19]. Despite this diversity, the database represents each of these experiments in one consistent format.

The uniform format shared by DREAM experiments can be thought of as having two main aspects: 1) meta-information about the experiment and 2) the experimental parameters and kinematic data. The meta-information includes information required to recreate or fully understand the experiment, such as the type of equipment used or the type of feedback a subject received. It also includes other important information associated with the experiment, such as the publication information. The other data is what scientists will spend most of the time analyzing. This recorded data would include the subject's kinematics, such as the hand position or the recorded forces, along with experimental parameters, such as the target location or the test condition of the reach. While experiments inherently record different types and amounts of data, the DREAM format requires aspects that are shared across datasets to be fully standardized. For example, we enforce using SI units throughout, eliminating a possible source of analysis error that was illustrated by NASA's multi-million dollar mistake [20]. Models and tools have access to both the data and the meta-information to generate predictions, display information, and make comparisons.

**Figure 3:**
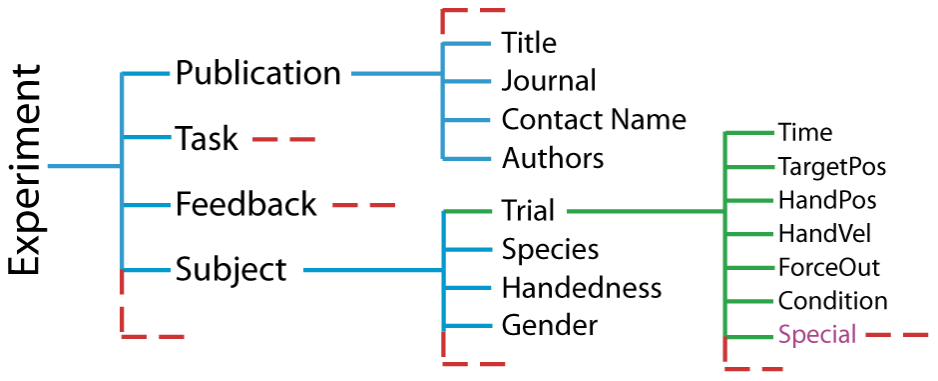
Hierarchical structure of experimental data in DREAM. Red dotted lines indicate that more fields could be present, but were omitted for space. Blue lines indicate meta-information; green lines indicate recorded experimental data. The contents of ‘Special’ are not standardized and can contain any arbitrary data. The ‘Subject’ field may also have a ‘Special’ field, depending on the experiment.

The format of datasets is hierarchical – technically a MATLAB structure – containing fields for the movement data and for the conditions under which the data were recorded (or predicted). At the top level, the ‘Subject’ field is an array of structures, indexed by subject number. Within the ‘Subject’ field is another array of structures named ‘Trial’, which contains the actual movement data (hand position, time trace, etc.). The fields in ‘Subject’ other than ‘Trial’ are subject-specific meta-information (age, gender, etc.). The top-level fields other than ‘Subject’ contain experiment-wide meta-information, including, among other things, task instruction, equipment used, and publication information. (See Fig. 3.)

This unified format would be useless if it were unable to accurately represent distinct experiments. To account for differences between experiments, the format representing experimental data needs to be sufficiently flexible. There are three main aspects to the flexibility of the DREAM data format. First, each experimental dataset can define arbitrary conditions (e.g. ‘null field’ or ‘catch trial’) depending on the procedure. Second, since not all experiments record the same data, some fields will not exist for some experiments. Each datum has a predetermined place where it should be stored and the presence of that field determines whether it was recorded or not. For instance, if a field called ‘HandVel’ is absent, then the hand velocity was not recorded for that particular experiment. If that field does exist, then the data stored within will be valid and in SI units (*m/s*). In rare instances, NaNs are used when small portions of data were missing, such as when a camera was blocked during recording. Third, the format can handle situations where data were recorded but do not fit into the pre-defined structure. Both the Subject and Trial structures have a field called ‘Special’ which is a structure that can store pertinent data that does not fit into the other predefined fields. Two examples of such data would be timestamps for auditory cues or peak tangential velocity. Because this format is arbitrarily extensible, any data in the ‘Special’ field is not standardized and this field cannot be expected to integrate with DREAM tools. The documentation, which is available for download with the rest of the DREAM project, elaborates further on each field and the specifics of the format.

To be added into DREAM, the minimum requirements for any experiment are that it involves reaching towards a target and tracking the hand (or limb) through space and time (joint angles or hand position or hand velocity). However, experiments are rarely fully described by only these criteria, so other data essential for analysis and recreation (e.g. forces or location of cursor feedback) needs to be present before we add the dataset into DREAM. As the database is currently structured, some upper limb movement experiments involve movement of a cursor towards a set of targets but cannot be included because the cursor position is determined by changing the hand (or wrist) posture or orientation, not a hand's location moving through space (such as [22], which involves wrist movements while holding smart phones). While interesting and pertinent research is happening outside these criteria, the increased database complexity necessary to include such sets makes it impracticable to include at present. However, in in the future, more types of experiment may be added.

### Models

**Figure 4:**
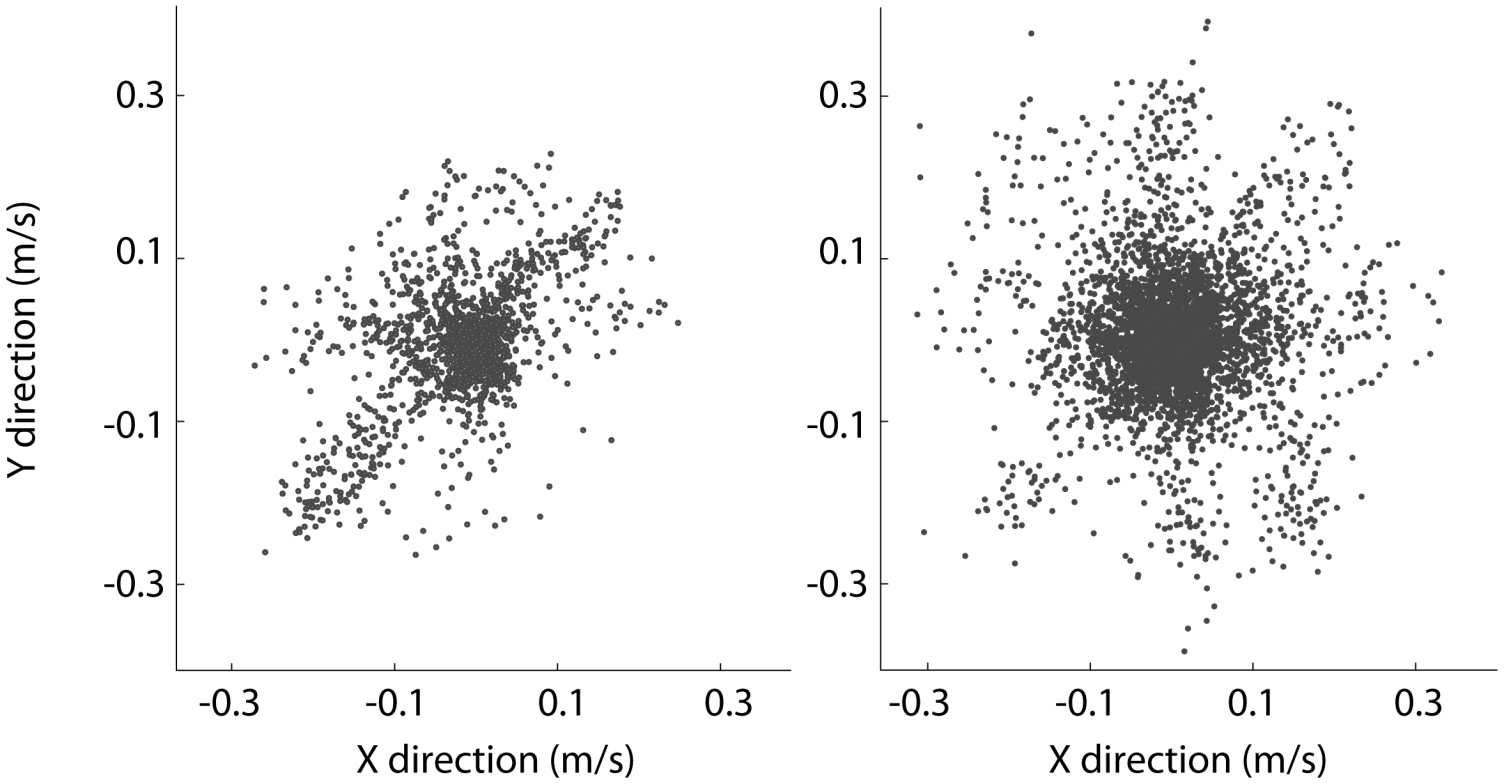
Preferred velocity of two neurons. The x–y velocity of the hand when a spike occurred. This shows two different neurons from two different monkeys performing the same center-out task.

**Figure 5:**
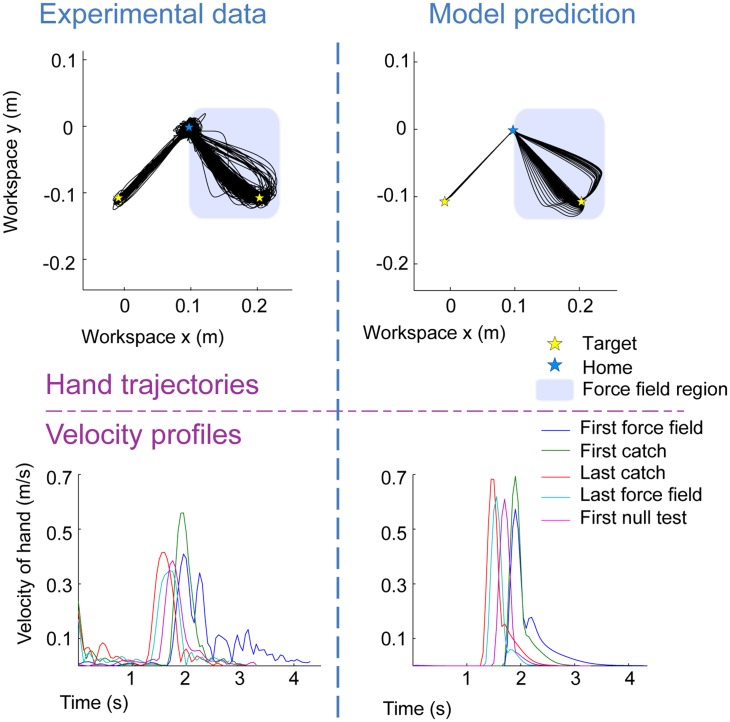
Two DREAM tools used to visualize experimental data and model predictions. Hand trajectories and velocity profiles from subject 13 of the Mattar_2007_e3 dataset, and the predictions of the same dataset using the Berniker_2011_m1 model. The force field region has been overlaid on the plot for clarity. For the velocity profiles, five reaches are displayed. One reach is the first force field the subject experienced (315°). We also show the velocity profile for the first and last catch trial the subject experienced (also at 315°), the last force field trial (315°) and the first null test trial, which involved reaching to the 225° target after learning the force field at the 315° target.

The minimum requirements for models are the same as they are for experiments. To be added into DREAM, a model must make a prediction of the hand moving to a target through space and through time. Models make predictions in the same space as experimental data, thus the format used to represent movement data (Fig. 3) is used for both predicted movements (model outputs) and recorded experimental data. Using the same format allows for the creation of tools that can be generalized (as shown in Fig. 4) or facilitate comparisons between models and data (as shown in Fig. 5).

**Figure 6:**
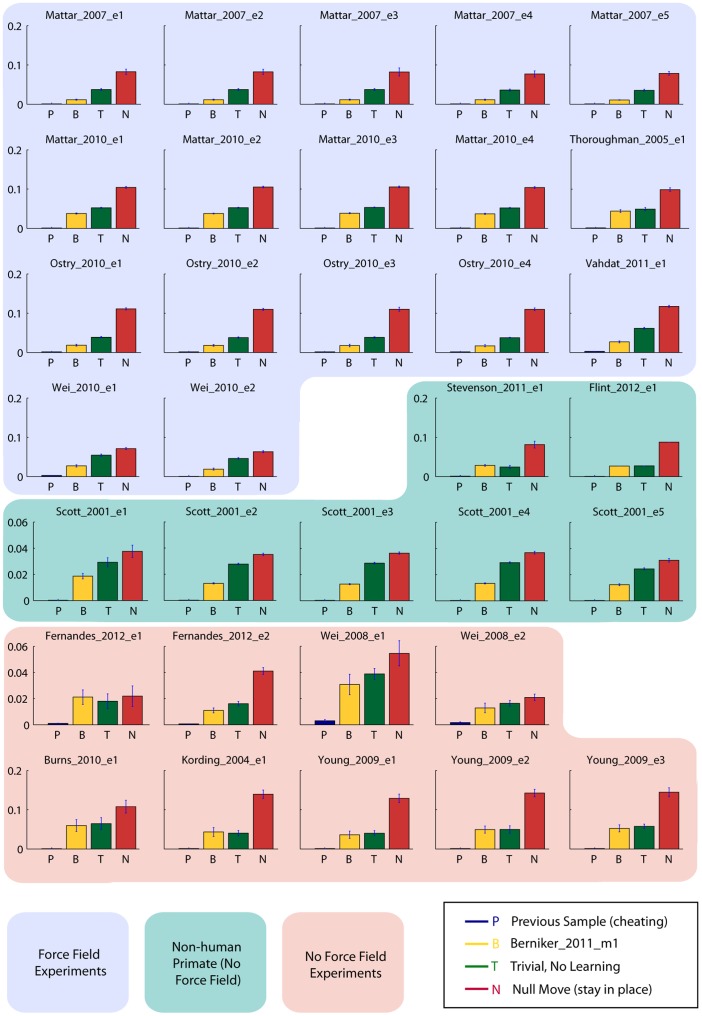
Mean Squared Error Averaged Across Subjects. The results of four separate models run against all experiments in DREAM. The Mean Squared Error (+/− STD) averaged across subjects for each experiment is reported.

Initially, experiment contributions have outpaced model contributions in DREAM. As such, we created trivial models; the predictions of these trivial models establish an envelope for the accuracy of real models. As a ceiling, we have created a cheating model (called Previous Sample in Fig. 6); it uses the actual trajectory to predict that the hand location at some time *t* will be what it was at the time *t−1*. In that sense, it will predict the precise reach trajectory, just shifted back one sample in time. Practically, a realistic model would never be able to out-perform this sort of model; the inherent variability of a person's reaching and the presence of outliers (subjects reaching to the wrong target) will allow this model to out-perform even the best “honest” models. Similarly, we have included a model that should perform worse than realistic models. This model assumes no movement (called Null Move); it will predict that the hand will stay in the start position throughout the reach. A model could theoretically do worse by moving away from the intended target, but such a model would likely have a bug in implementation (e.g. a sign problem or wrong assumptions).

We also have a non-learning model that reaches directly toward a target (called Trivial No Learn). It does not consider visual feedback, thus any cursor perturbations will not affect it. It also does not correct for position errors, thus can be pushed of course if a force field is present. Such a model serves as a good model to try to beat; it will be a reasonable approximation of what a human might do. Because it ignores visual perturbations, it will be much closer to the target than a human subject would when visual perturbations are present. Because it does not account for position errors, it will be much further from the target in the presence of a non-corrective force field. DREAM also has a force-field learning model from [21], which also ignores visual feedback, and will examined further in the Results section.

These models should be able to generate predictions for a broad sampling of datasets. However, some models might require certain specific pieces of information. For instance, if a model required EMG data, it could be added into DREAM, but it would not be able to make predictions for the majority of experiments. DREAM also contains, for teaching purposes, a neural decoder that uses principle component analysis and a least squares covariance to make kinematic predictions. This is useful for scientists interested in decoding from a brain-machine interface perspective. Of course, this model works only for datasets where spike timestamps were recorded.

When models from the database are run using DREAM's tools, they are passed a dataset that includes both the movement data and the meta-information. The model is responsible for parsing out the values it needs; because of the standardized format, models can look for the data in one location and use default values (or fail gracefully) if a particular piece of data is missing or is unexpected (e.g. NaN). It then makes a prediction and puts the data in the correct structure (mimicking the experiment data format). The models are stored as .m files, formatted to accept arguments as defined by DREAM's Run Model tool (Fig. 1). Models that output predictions appropriately allow the DREAM tools to treat the predictions just as they would an experimental dataset; thus, the same DREAM tool can plot the velocity profiles of experimental data or of predicted data (for an example, see Fig. 5).

### Tools

The DREAM tools operate much like a MATLAB toolbox; they are a suite of tools designed around a central idea. Users can use these tools to interact with DREAM datasets and models in a variety of ways. Examples of analyses and tool outputs are described in the Results section below.

A scientist who wanted to create or add new models, tools, underlying utility functions, or model comparison metrics will need to learn the DREAM format. He or she could use current tools as a guide and could refer to the appropriate section in the user manual. Documentation regarding tool syntax, purpose, and creation can be downloaded from http://crcns.org/data-sets/movements/dream/documentation. To integrate with and employ the utility functions and tools of DREAM, he or she would also need to understand the order of arguments and syntax used by the rest of DREAM. The benefit of taking the time to create new tools or metrics is that new tools can be shared with all users of DREAM.

While it is desirable that each tool would work for all datasets, some DREAM tools apply to only a subset of DREAM datasets. For instance, we have a tool that plots the preferred direction of neurons (for an example, see Figure 4). Since not all experiments record neural spike timestamps, this tool will only work for datasets that include spike information (e.g. [15] and [16]). However, some tools, like an animation tool, have been written to work for all experiments.

Because of the shared data format, tools can take inputs of model predictions or experimental data interchangeably. Thus, we can use the same tools to make qualitative comparisons between data and predictions (for an example, see Fig. 5). DREAM also has tools that are meant to compare models against each other. By measuring each model according to the same metric, we can see which performs better for which experiments (for an example, see Fig. 6). The Results section below will show the outcome of using some of these tools.

## Results

To showcase the possible uses of DREAM, we have included examples of using DREAM tools to analyze DREAM data and models in a few different ways. Scientists ought to be able to expand and adapt these tools to their own uses. But as displayed below, these tools would be useful for experimentalists who want to understand how their data relates to models, for modelers who want to test their theories, and for educators who want to help students better understand reaching experiments, models, and data analysis.

### Using DREAM Tools to look at data

One of the tools in DREAM allows a user to plot the preferred direction of neurons. This tool will work only if timestamps of spikes are recorded in the neuron structure as defined in the documentation of DREAM. Data from two such publications have been included in DREAM: [15] and [16], with DREAM IDs of “Flint_2012_e1” and “Stevenson_2011_e1” respectively. (The ‘e1’ at the end means the data is the first experiment associated with the publication, as defined on the download page of the dataset.)

Figure 4 shows the results of this tool. A point is plotted every time the neuron fired during the experiment. The point is placed at the corresponding x–y value of the velocity when the spike occurred. In these experiments, the monkey moved a handle in a two dimensional plane that moved a cursor on a screen in front of the animal. The task was a center-out reach with 8 targets equally spaced (every 45 degrees) around the edge of a circle with a 10 cm radius.

### Using DREAM Tools to compare data and models

To compare experimental results with model predictions, we can plot position and velocity traces using DREAM tools. To illustrate this feature, we will use an example dataset and model from the database. For an example dataset, we use data from subject 13 of an experiment designed to look at generalization within the workspace [12]. The dataset is called “Mattar_2007_e3” in DREAM. At the beginning of the experiment, the subject started by making baseline movements to a target located at 225° (see Fig. 5, top left). They then reached to another target, located at 315°, during a training phase in a null field and a testing phase that had a velocity-dependent curl field with catch trials. Finally they reached to the original target (225°) in a null field again.

For our example model, we use a generic force field adaptation model (DREAM ID: Berniker_2011_m1) that uses a 4-dimensional linearization of the limb in space [21]. The model was designed to explain force field adaptation for center out reaches. Parameters were not specifically tuned for performance, but some experiment-specific values, such as the duration of reach, were used to help make useful predictions.

After loading the dataset into MATLAB, we can use DREAM tools to examine the experimental data, run models to generate predictions, and compare the predictions with the experimental results. Figure 5 shows the trajectories of the subject's hand for all reaches in the experiment alongside the trajectories that the model predicted. Importantly, the hand trajectories of both the model prediction and the experimental data were plotted using the same DREAM tool. Because the reaches to the 225° target were performed in a null field, they are very direct. By comparison, reaches to the 315° target occur within a force field (with some catch trials) and therefore take less direct paths.

Another tool in DREAM plots the hand's velocity profile. In Figure 5, we show the velocity profiles of five reaches of interest. Since we used the same tool to plot both the model results and the experimental data, we can easily compare the two. The model's prediction is similar in a number of ways to the experimental data but also clearly differs, e.g. with respect to the smoothness of the velocity.

Comparing one model to one experiment in this way can give us insights into what experimental conditions make the model more or less accurate. By examining on a trial by trial basis, we can learn where model predictions might break down. But we are also interested in assessing whether certain models perform well for some experiments and poorly for others. By running all models against all datasets, we can get a sense of how models compare and what sort of experiments each does well at describing.

### Using DREAM Tools to compare models

We used four models described in the methods section to make predictions: a “null move” model, a non-learning model that tries to move straight to the target (without correcting for visual or physical perturbations), the force field adaptation model from Figure 5 and a “cheating” model that predicts the previous point in the reach as the next point in the reach. After generating predictions with the Run Model tool, we used another DREAM tool to calculate mean squared error (MSE) of each model. The mean squared error tool computes the MSE between the prediction and the actual data and, using a third DREAM tool, we can plot the MSE for each trial or across subjects or for the whole experiment. In Figure 6 we show the averages of all subjects within each experimental dataset.

Since it is designed to describe force field experiments, the Berniker_2011_m1 model performs well for experiments that involved force fields [2], [6], [11], [12], [18] – its average error was about half (51%) that of the Trivial No Learning model. When experiments did not involve force fields, both the Trivial No Learn and the Berniker_2011_m1 move directly toward the target. For these, the Berniker_2011_m1 model had about 81% of the error of the Trivial No Learn model. Also, both models perform very poorly (very similar to Null Move) for the Fernandes_2012_e2 experiment, which gradually introduced a 30 degree visual rotation. Since both the Trivial No Learn and the Berniker_2011_m1 model ignore visual feedback, they predict straight reaches that move directly toward the target. The actual data shoes that subjects do respond to the cursor rotation and make reaches that are rotated away from the target. The models do not correct for this cursor rotation; thus they make predictions that terminate closer to the displayed target than the actual reaches did.

### Adding data into DREAM

Because each submission we received had a unique organization, we wrote MATLAB functions that read the data in (from .mat or .txt files) and save it according to the correct DREAM format. These functions allowed us to parameterize the data sifting process. We can change the downsampling rate or the value used to convert cursor screen coordinates to meters, allowing us to quickly make changes if a user requests a non-downsampled dataset or if an error is discovered in cursor conversion. We can also reuse some of the functions when we get submissions similar to datasets we have already added; this can reduce the required sifting time when labs make multiple submissions.

## Discussion

### Major Contributions of DREAM

With the available datasets and tools, DREAM enables comparing models against multiple datasets. By analyzing how models perform against different datasets, scientists can explore strengths and weaknesses of certain modeling approaches under certain conditions. Experimentalists can also use DREAM to explore how their data relates to models. Educators can use DREAM to aid in the learning environment, providing a way for students to interact with and analyze data. Lastly, DREAM expands the availability of data, giving access to those who might be transitioning into the field, to those for whom obtaining data is difficult, or to those who would like to analyze data from a range of experiments.

### Data Availability

The process of sifting the data and putting it into the DREAM format sometimes revealed problems in the dataset. While trying to match what we saw in the data with what is reported in the paper, we occasionally uncovered discrepancies. Sometimes we discovered figures which omit information or that the number of trials of a certain condition was different than what was reported in the publication. We then went back to the data providers, asking them to explain. On rare occasions, we come to an impasse (part of the data cannot be found or the target of the reach was not saved) and had to decide whether to include the data, making inferences where appropriate, or to refrain from loading the data into DREAM. As an example of inferring data, one dataset omitted target position. But while it may not have been explicitly recorded, velocities and hand positions traces can give a good indication of where the target was. In all our actions, we worked to maintain the integrity of the dataset, so that the files available for download were maximally useful.

After the sifting was finished, the new dataset was saved with a unique identification tag and then uploaded. The unique identification was created using the last name of the first listed author, the year of publication, and a number indicating different experiments present in the publication. If we received a dataset that is not attached to a publication, we can still use the last name of the contributing author and the year it was added. Often one publication looks at a few different experimental conditions. As such, we divide the publication's data into a number of experiments. These are defined in an associated info.txt file explains the divisions (and any other oddities about the data). On the dataset's download page, the info.txt file and a PDF of the publication are made available.

In general, we found scientists were willing to share data. Some granting agencies and journals hold data sharing as a condition of publication and funding (see http://www.nature.com/authors/policies/availability.html, http://grants.nih.gov/grants/policy/data_sharing/data_sharing_guidance.htm) and some granting agencies, e.g. the NSF provide special grants for data sharing (http://www.nsf.gov/pubs/2011/nsf11505/nsf11505.htm). However, it seemed that most scientists we contacted were willing to make their data public simply to make the data useful to the community. However, there were a few who held back their data, either for personal reasons or because of doubts about the generality of the conclusions.

### Applications for and Limitations of DREAM

The tools allow us to examine the trajectories of the hand and the velocity profiles of movements. Using these, a person can quickly become familiar with data they have not seen before – the animation tool is also especially useful in this regard. DREAM tools allow a user to readily identify outliers, selectively examine data (e.g. by looking at only catch trials), and make qualitative model/experiment comparisons. The tools also allow for quantitative comparisons of models. Using DREAM promises to be useful for students entering the field to learn about current research and to practice data analysis. In fact, we extensively used the database as part of the 2012 Summer School in Computational Sensory-Motor Neuroscience (CoSMo 2012, online at http://www.compneurosci.com/CoSMo2012/) and it was rated as very useful by the students. The database allowed us to enhance the student experience, and we anticipate its use growing as people come to understand what it is able to do.

One area in which DREAM is lacking is in the number of model submissions. In the early stages of this project, it has been necessary to focus on experimental dataset inclusion; model inclusion was postponed because some models required access to meta-information that was not recorded in certain experiments. While we do make every effort to include all the information we can from the experiment in the database, there may be some information a model might want – such as subject meta-information (gender, height, age) or behavioral information (gaze direction) – that wasn't recorded in the experiment and is therefore not contained in the database. However, as more experiments are being added to DREAM, there is a greater chance that the relevant information is available in a reasonable number of datasets. We anticipate broader exposure to the community will increase model submission. Summer schools such as CoSMo 2012 will teach attendees how to use the database, broadening its exposure. We also anticipate having model competitions that use the database, which will also increase model contributions.

One challenge we have faced is that some of the experiments in DREAM do not include all the data we would like or all that is reported in the published paper. For instance, some experiments monitor position with a camera that is sometimes blocked during the experiment. For other datasets, target locations or training data were not recorded. In some cases, some of the data used in the analysis of the paper has been lost. When appropriate, we have interpolated what we can, but sometimes we need to omit data or leave place holders (NaNs). It is our goal to make the dataset as useful as possible, inferring target position if appropriate, but most importantly, to retain the data's integrity.

### Appropriateness of DREAM

DREAM is not unique in its approach of collecting experimental data into one database. Many other databases (http://neuinfo.org/nif/nifgwt.html, http://en.wikipedia.org/wiki/List_of_neuroscience_databases) are very useful to scientists. However, two aspects of DREAM make it stand out. First, by focusing on reaching, DREAM fills a gap in the resources available to movement scientists. Second, unlike most databases, DREAM is curated. Although DREAM's use of kinematic reaching data from different labs offers new analysis possibilities, its potential really lies in standardization of data. By standardizing, analyses become streamlined and broader questions can be asked. This can best be facilitated with a curated database.

Despite the work done so far, there are still ways to make the DREAM project more useful. A feature we would like to add is having tools that reproduce figures from specific publications. There is also potential for further development in increasing the number and type of metrics used to compare models, like deviation from a straight path or end point error. More tools could be written to allow for other kinds of analysis, as well. These tools could then be incorporated into the downloadable DREAM tools in future releases. And, of course, we'd like to add more models and continue to add datasets. Our goal is for the DREAM project to grow organically; as members in the community begin using it more, development can be focused on features of DREAM that would be most helpful.

Some might argue that this project places restrictions on scientists; for instance, by choosing MATLAB as our environment, we reduce the number of people who can interact with the database. However, an environment must be chosen; and while perhaps not catering to every potential user, we chose MATLAB because we believe it makes the database accessible to the largest subset of scientists in the field. While an open-source solution (such as Octave) was initially considered, preliminary inquiries with potential users indicated MATLAB would be more useful. We also did not want to take on the administrative burden of developing in two formats. The database was designed to incorporate new tools as they were developed; if labs have analyses that are written in other languages, we encourage them to port them over to MATLAB to share with other users of DREAM.

Another argument on restriction revolves around standardizing data format. Does creating a standard stifle flexibility and create restrictive policies? Whenever standards are created, certain boundaries need to be set. Indeed, we included an example of a dataset that must be omitted. We are aware there are some valid reasons to not standardize – for instance, the overhead of the format structure is non-trivial for certain applications – however, creating a place for data to be collected and shared is a good step in reducing unnecessary duplication (and perhaps doing good verification), and a way of discussing standard paradigms shared by the community.
